# Comparative bactericidal activity of four fluoroquinolones against *Pseudomonas aeruginosa* isolated from chronic suppurative otitis media

**DOI:** 10.1186/s12901-015-0018-9

**Published:** 2015-10-14

**Authors:** Katsuhisa Ikeda, Shigeki Misawa, Takeshi Kusunoki

**Affiliations:** Department of Otorhinolaryngology, Juntendo University Faculty of Medicine, 2-1-1 Hongo, Bunkyo-ku, Tokyo 113-8421 Japan; Microbiology Laboratory, Juntendo University Faculty of Medicine, 2-1-1 Hongo, Bunkyo-ku, Tokyo 113-8421 Japan

**Keywords:** Fluoroquinolone, *Pseudomonas aeruginosa*, Chronic suppurative otitis media, Bactericidal activity, Minimum inhibitory concentration

## Abstract

**Background:**

The aim of the present study was to evaluate the bactericidal activity of four new fluoroquinolones against current isolates of *Pseudomonas aeruginosa* from the patients with chronic suppurative otitis media (CSOM).

**Methods:**

We examined bactericidal activity of four types of fluoroquinolones, garenoxacin (GRNX), levofloxacin (LVFX), ciprofloxacin (CPFX) and sitafloxacin (STFX) against current isolates of *P. aeruginosa* (50 strains).

**Results:**

STFX exhibited the most potent activity of both MIC_50_ and MIC_90_, followed by CPFX, LVFX, and GRNX. The number of GRNX-resistant strains was significantly greater than those of LVFX, CPFX, and STFX (*P* < 0.05).

**Conclusion:**

STFX showed the most potent activity against *P. aeruginosa* for recent pathogens recovered from CSOM as compared with the others, suggesting that the clinical application of topical STFX would be useful to prevent the emergence of resistant mutants of *P. aeruginosa*.

## Background

Chronic suppurative otitis media (CSOM) is defined as tympanic membrane perforation with ear discharge or otorrhea present continuously for at least 2 weeks [1, 2], and can result in thickening of the middle ear mucosa and mucosal polyps. CSOM continues for months or years with increasing hearing impairment; it can lead to life-threatening infective complications [3, 4]. The commonly isolated microorganisms are *Pseudomonas aeruginosa* and *Staphylococcus aureus*; *P. aeruginosa* has been particularly implicated in the causation of bony necrosis and mucosal disease. Newer third- and fourth-generation fluoroquinolones often possess excellent in vitro activity against the most common respiratory pathogens [5].

**Fig. 1:**
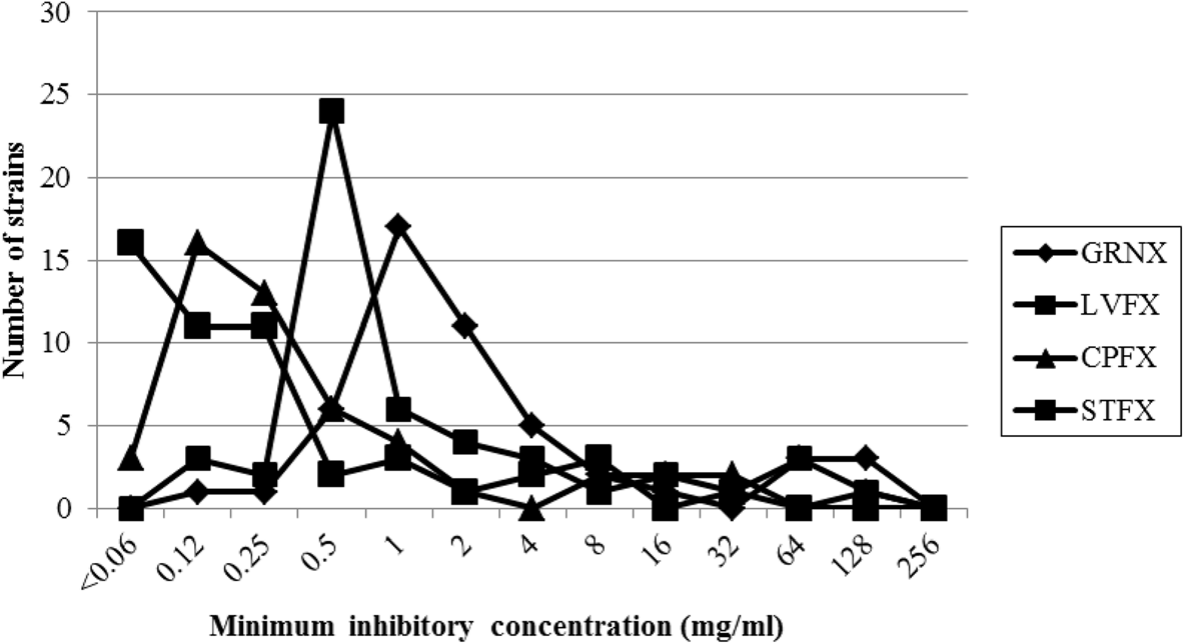
The distribution of minimum inhibitory concentrations of *Pseudomonas aeruginosa*

In the present study, we evaluated the bactericidal activity of four new fluoroquinolones against current isolates of *P. aeruginosa* from patients with CSOM.

## Methods

We collected *P.aeruginosa* isolated from clinical specimens taken from the middle ear perforation under a microscope in patients with CSOM at the Department of Otorhinolaryngology, Juntendo University Hospital from January in 2010 to March in 2013. Sampling was random and continuous and those who had recently used local or systemic antibiotics were excluded. The specimens for all bacterial culture were promptly transported in culturette tubes kept moist with Stuart’s bacterial transport medium. The study was approved by the ethics committee of the Juntendo University Faculty of Medicine. The informed consent was not required since all data were collected as part of routine diagnosis and treatment, and were retrospectively analyzed. Total number of strains of *P.aeruginosa* was 50. The subjects from whom *P.aeruginosa* was recovered were 32 males and 18 females ranging in age from 1 to 90 years (mean age 51.7 years).

For antimicrobial susceptibility testing, we measured the minimum inhibitory concentration (MIC) by the broth microdilution method, complying with the Clinical and Laboratory Standards Institute (CLSI) standard [6]. Drug-containing agar plates were inoculated with 5 μl of each specimen and the plates were incubated for 16–20 h at 35 ± 2 °C. The MIC was defined as the lowest drug concentration that prevented visible growth of the bacteria. The MIC_50_ and MIC_90_ were defined as the MICs at which 50 % and 90 % of isolates are inhibited, respectively. The strains were classified according to the CLSI criteria [7]. Sensitivity to four types of fluoroquinolones, garenoxacin (GRNX), levofloxacin (LVFX), ciprofloxacin (CPFX) and sitafloxacin (STFX), was tested and was classified according to the CLSI criteria [8]. Namely, the *P.aeruginosa* strains were classified as GRNX-sensitive (MIC ≤ 1 μg/ml), −intermediate (MIC = 2 μg/ml), resistant (MIC ≥ 4 μg/ml) strains; LVFX-sensitive (MIC ≤ 2 μg/ml), −intermediate (MIC = 2 μg/ml), resistant (MIC ≥ 8 μg/ml) strains; CPFX-sensitive (MIC ≤ 1 μg/ml), −intermediate (MIC = 2 μg/ml), resistant (MIC ≥ 4 μg/ml) strains; STFX-sensitive (MIC ≤ 1 μg/ml), −intermediate (MIC = 2 μg/ml), resistant (MIC ≥ 4 μg/ml) strains.

Statistical analyses were evaluated using StatMate IV for Windows. Chi-square test　was used to compare the susceptibility for 4 fluoroquinolones. Results were considered to be significant if the *P* values were less than 0.05.

## Results

The Fig. 1 shows the distribution of MIC for the 4 fluoroquinolones against *P. aeruginosa*. Among the 4 fluoroquinolones tested here, STFX exhibited the most potent activity at both MIC_50_ and MIC_90_, followed by CPFX, LVFX, and GRNX (Table 1). Table 2 shows a summary of the fluoroquinolone-sensitive, −intermediate, −resistant strains of *P. aeruginosa*. The number of GRNX-resistant strains was significantly greater than those of LVFX, CPFX, and STFX (*P* < 0.05).

**Table 1:** MIC_50_ and MIC_90_ of *Pseudomonas aeruginosa*

	MIC_50_ (μg/ml)	MIC_90_ (μg/ml)
GRNX	1	64
LVFX	0.5	32
CPFX	0.25	16
STFX	0.12	4

**Table 2:** The susceptibility of *Pseudomonas aeruginosa* to fluoroquinolones

	Number of strains (%)		
	Susceptible	Intermediate	Resistant
GRNX	25 (50)	11 (22)	14 (28)
LVFX	39 (78)	3 (6)	8 (16)
CPFX	42 (82)	1 (2)	7 (14)
STFX	43 (86)	1 (2)	6 (12)

## Discussion

This is the first report to compare the bactericidal activity of 4 fluoroquinolones, namely GRNX, LVFX, CPFX, and SIFX, against *P. aeruginosa* recovered from CSOM. A nationwide surveillance of antimicrobial susceptibility of bacterial lower respiratory pathogens from patients in Japan between 2006 and 2007 reported that, in a total 103 *P. aeruginosa* strains, CPFX among 6 fluoroquinolones showed the most potent activity and that the other fluoroquinolones showed strong activity but were suggested to have met with partial resistance [9]. However, this report did not include the antimicrobial susceptibility of STFX. The high sensitivity rate for *P. aeruginosa* to CPFX in the present study is well comparable to that reported in recent studies [10–15], whereas there are no previous data available on the susceptibility of *P. aeruginosa* isolated from CPOM to GRNX, LVFX, and STFX. The present study clearly demonstrated that STFX showed the most potent activity against *P. aeruginosa* for recent pathogens recovered from CSOM as compared with GRNX, LVFX, and CPFX.

The isolation rate of *P. aeruginosa* strains resistant to antibiotics has increased recently, making it more difficult to select adequate antibiotics. Resistance to fluoroquinolones in the present study ranged from 12 to 28 %, although the MIC of STFX was less than 32 μg/ml. Two major mechanisms [16, 17] may lead to fluoroquinolone resistance to *P. aeruginosa*; i) modification of the primary target (DNA gyrase) and secondary target (topoisomerase IV) by point mutations in gyrA/gyrB and parC/par genes, respectively, and ii) four efflux systems identified in *P. aeruginosa*.

In the absence of systemic infection or serious underlying disease, first line pharmacologic treatment for most patients with CSOM usually entails oto-topical fluoroquinolones such as ofloxacin and CPFX. High concentrations are pharmaco-dynamically important for antibiotics known to have a concentration-dependent mechanism of action such as fluoroquinolones. Consequently, the concentration of delivered topical fluoroquinolones seems always well above the MIC of *P. aeruginosa*, making the emergence of bacterial resistance extremely unlikely [18]. Moreover, the present study may encourage the clinical application of topical STFX as a treatment for CSOM in order to prevent the emergence of resistant mutants of *P. aeruginosa*.

## Conclusion

STFX showed the most potent activity against *P. aeruginosa* for recent pathogens recovered from CSOM as compared with the others, supporting the clinical application of topical STFX in order to prevent the emergence of resistant mutants of *P. aeruginosa*.

## Abbreviations

CSOM: Chronic suppurative otitis media
LVFX: Levofloxacin
GRNX: Garenoxacin
CPFX: Ciprofloxacin
STFX: Sitafloxacin
MIC: Minimum inhibitory concentration
CLSI: Clinical and Laboratory Standards Institute
